# Promising Tropical Fruits High in Folates

**DOI:** 10.3390/foods8090363

**Published:** 2019-08-26

**Authors:** Lisa Striegel, Nadine Weber, Caroline Dumler, Soraya Chebib, Michael E. Netzel, Yasmina Sultanbawa, Michael Rychlik

**Affiliations:** 1 Chair of Analytical Food Chemistry, Technical University of Munich, 85354 Freising, Germany; 2 Queensland Alliance for Agriculture and Food Innovation, The University of Queensland, Coopers Plains, QLD 4108, Australia

**Keywords:** folate, tropical fruits, subtropical fruits, vegetables, indigenous food, stable isotope dilution assay, LC-MS/MS

## Abstract

As the popularity of tropical fruits has been increasing consistently during the last few decades, nutritional and health-related data about these fruits have been gaining more and more interest. Therefore, we analyzed 35 samples of tropical fruits and vegetables with respect to folate content and vitamer distribution in this study. The fruits and vegetables were selected by their availability in German supermarkets and were grouped according to their plant family. All fruits and vegetables were lyophilized and analyzed by stable isotope dilution assay (SIDA) and liquid chromatography mass spectrometry (LC-MS/MS). The results vary from 7.82 ± 0.17 µg/100 g in the horned melon to 271 ± 3.64 µg/100 g in the yellow passion fruit. The yellow passion fruit is a good source for meeting the recommended requirements, as just 110 g are needed to cover the recommended daily intake of 300 µg folate for adults; however, longan fruits, okras, pete beans, papayas, mangos, jack fruits, and feijoas are also good sources of folates. In conclusion, the study gives a good overview of the total folate content in a broad range of tropical fruits and vegetables and shows that some of these fruits definitely have the potential to improve the supply of this critical vitamin.

## 1. Introduction

Tropical or exotic fruits are usually known as fruits which grow in tropical or subtropical climates [1]. Besides well-known fruits such as banana, kiwi, or pineapple, which have been available in supermarkets all over the world for quite some time, more and more tropical fruits have become increasingly popular in recent years [2]. These tropical fruits include for example pitaya, mango, and jack fruit [3,4]. Their popularity is mainly due to the diversity of fruits regarding the exotic taste and smell and unusual shape and colors. Optimized transport systems such as insulated and refrigerated containers ensure the optimum temperature for the tropical fruits during long journeys or daily flights, guaranteeing a short transport time and facilitating exportation worldwide [4,5]. Due to their growing popularity, these fruits are also becoming increasingly interesting in terms of their ingredients and nutritional benefits [6]. In general, regular consumption of fruits can be preventive against different diseases like heart disease, diabetes type II, and obesity and can also provide a significant amount of vitamins, minerals, fiber, and phytochemicals such as polyphenols and carotenoids [1,7,8]. Apart from the highly abundant phenolic compounds and the antioxidant properties of exotic fruit, their other bioactive compounds such as vitamins should be investigated. So far, some exotic fruits have already been analyzed with respect to their total folate content [9,10,11], but there is still a gap regarding folate data in indigenous food. With respect to the influence of different environmental factors, such as for example climate, geographical variations, and seasons [11,12] on the total folate content, it is fundamental to provide a broader data basis.

Folate is an important vitamin in human metabolism. It functions as a coenzyme and is needed for one carbon transfers and synthesis in nucleotide and amino acid pathways [13,14]. Folate cannot be synthesized by the human body, and therefore must be supplied by food or supplements. As there is a tendency towards suboptimal intake, particularly in Europe the risk for some diseases has increased [15]. For young women of childbearing age in particular it is important to get enough folate since a deficiency is associated with a higher risk for neural tube defects in newborns [16]. Therefore, supplementation with folic acid is highly recommended before and during pregnancy. An insufficient intake of folate can also cause other chronic diseases such as Alzheimer’s, autism, and cardiovascular disease [17,18,19,20]. This is attributed to a limited conversion of homocysteine to methionine, which leads to a consistent increase of the homocysteine level [17]. Consequently, it is crucial to have a reliable analytical method to provide accurate food-folate data, which can be used for dietary recommendations.

Liquid chromatography mass spectrometry (LC-MS/MS) methods using stable isotope dilution assay (SIDA) have stood the test of time over conventional methods such as microbiological assays and high performance liquid chromatography (HLPC-UV) methods. The use of an isotopologic internal standard compensates for analyte degradation, analyte conversion, or losses during extraction as well as matrix effects and ion suppression during LC-MS/MS measurements [21]. As the group of folates consists of different vitamers which show different stabilities and conversion reactions, SIDA is a solid and specific method for folate analysis and for the determination of the folate pattern in food. In the following study a broad range of tropical fruits and vegetables were analyzed regarding their total folate content and vitamer profiles. The investigated vitamers were folic acid (PteGlu), tetrahydrofolate (H_4_folate), 5-methyltetrahydrofolate (5-CH_3_-H_4_folate), 5-formyltetrahydrofolate (5-CHO-H_4_folate), and 10-formylfolic acid (10-CHO-PteGlu). The analyzed tropical fruit samples were of mango, pitaya, papaya, kaki, guava, feijoa, okra, horned melon, lucuma, salak, tamarillo, lonkong, longan, passion fruit, cherimoya, tamarind, pete beans, Chinese jujube, prickly pear, and jack fruit. All fruits were analyzed by SIDA and LC-MS/MS according to the method of Striegel et al. [22].

## 2. Materials and Methods

### 2.1. Samples

A broad range of tropical and subtropical fruits and vegetables was investigated in the present study. All fruits were purchased at the eating ripe stage in local supermarkets near Munich, Germany except feijoa, which was supplied by Produce Art, Salisbury, QLD, Australia. The countries of origin where possible are listed in Appendix A. The fruits were immediately cut into small pieces and lyophilized. All fruits were thoroughly blended with a commercial hand mixer and extracted.

### 2.2. Sample Extraction

The sample extraction was performed as described previously with slight modifications [22]. Briefly, 50–100 mg of initially homogenized fruit and vegetable samples were weighed into Pyrex bottles and equilibrated with 10 mL of buffer for extraction (200 mmol/L 2-(*N*-morpholino)ethanesulfonic acid hydrate (MES), 114 mmol/L ascorbic acid, and 0.7 mmol/L DL-dithiothreitol (DTT), pH 5.0). Internal standards [^13^C_5_]-PteGlu, [^13^C_5_]-H_4_folate, [^13^C_5_]-5-CH_3_-H_4_folate, and [^13^C_5_]-5-CHO-H_4_folate were added to the samples in amounts adjusted to the expected content of analytes to fall in the given calibration range. For deconjugation, 2 mL of chicken pancreas (1 g/L in 100 mmol/L phosphate buffer, 1% ascorbic acid) and rat serum (400 µL–800 µL, amount adjusted to receive complete deconjugation) were added to the samples and were incubated overnight at 37 °C. Samples were purified by strong anion-exchange (SAX, quaternary amine, 500 mg, 3 mL) solid–phase extraction (SPE) using buffer for equilibration (10 mmol/L phosphate buffer, 1.3 mmol/L DTT) and 2 mL buffer for elution (5% sodium chloride, 1% ascorbic acid, 100 mmol/L sodium acetate, and 0.7 mmol/L DTT).

### 2.3. Instrumental Conditions

All LC and MS conditions were as previously described [22]. Briefly, the concentration and purity of unlabeled analytes, which were prepared new before each extraction, were determined using a Shimadzu HPLC/DAD system (Shimadzu, Kyoto, Japan) equipped with a reversed phase column (C18 EC, 250 × 3 mm, 5 µm, 100 Å, precolumn: C18, 8 × 3 mm, Machery-Nagel, Düren, Germany). The mobile phases for gradient solution were (A) 0.1% acetic acid and (B) methanol.

LC-MS/MS measurements of samples were performed on a Shimadzu Nexera X2 UHPLC system (Shimadzu, Kyoto, Japan) equipped with a Raptor ARC-18 column (2.7 µm, 100 × 2.1 mm, precolumn: 2.7 µm, 5 × 2.1 mm, Restek, Bad Homburg, Germany). The mobile phases for the binary gradient consisted of (A) 0.1% formic acid and (B) acetonitrile with 0.1% formic acid at a flow rate of 0.4 mL/min.

The LC was interfaced with a triple quadrupole ion trap mass spectrometer (LCMS-8050, Shimadzu, Kyoto, Japan). Samples were measured in the ESI positive mode as previously described [22].

### 2.4. Method Validation

The validation data of the method are described in a previously published paper [22]. Briefly, for determining the limits of detection (LOD) and quantification (LOQ) we used a folate-free matrix of sugar and pectin. The major ingredients of fruits and vegetables are dietary fibers and sugar, which are similar to the used matrix for validation. Therefore, the validation is also deemed valid for tropical fruits and vegetables. The LOD and LOQ values for PteGlu were 0.33 µg/100 g and 0.96 µg/100 g, for H_4_folate they were 0.25 µg/100 g and 0.76 µg/100 g, for 5-CH_3_-H_4_folate they were 0.17 µg/100 g and 0.51 µg/100 g, and for 5-CHO-H_4_folate they were 0.32 µg/100 g and 0.93 µg/100 g, respectively. Recoveries of all analytes were in a range between 81.9% and 114%. Inter-injection precisions were between 1.92% and 4.46%, intra-day precisions were between 2.44% and 2.74%, and inter-day precisions were between 3.04% and 5.06%. Quantitation of 10-CHO-PteGlu was performed using [^13^C_5_]-5-CHO-H_4_folate as internal standard. The LOD and LOQ were estimated using the response factor of 5-CHO-H_4_folate as a reference value. 10-CHO-PteGlu proved to be detectable more sensitively than 5-CHO-H_4_folate and we calculated an estimated LOD of 0.14 µg/100 g and a LOQ of 0.40 µg/100 g.

## 3. Results and Discussion

Different tropical foods, among them various fruits and several vegetables, were analyzed for their total folate content and vitamer profiles based on fresh weight. The most abundant folate vitamers in food, namely 5-CH_3_-H_4_folate, 5-CHO-H_4_folate, 10-CHO-PteGlu, H_4_folate, and PteGlu were determined. The fruits and vegetables analyzed in this study were selected according to their availability in German supermarkets and food markets. In this paper, the fruits were grouped according to their plant family whenever possible.

### 3.1. Folate Content in Mango

Various mango fruits were analyzed for their total folate content as well as their vitamer distribution (Table 1). Mango (Anacardiaceae, *Magnifera indica*) is originally from India, however, in the course of time mangos have been cultivated in the tropical forests worldwide. We analyzed five different varieties of mango and found total folate contents ranging between 55.8 ± 0.73 and 74.5 ± 2.09 µg/100 g. The predominant vitamer was 5-CH_3_-H_4_folate (88.3–90.9%), followed by low amounts of 5-CHO-H_4_folate (4.62–6.30%). Akilanathan et al. [11] examined different varieties of mangos and found similar, partly higher folate contents of between 60.0 and 138 µg/100 g. The highest folate content was found in the smallest and unripe fruit. Our findings were similar to those of Akilanathan et al., with the highest folate content in the fruit with the smallest size and lower folate contents in fruits with bigger sizes. Further investigations are warranted to elucidate the relationship between fruit size and folate content.

### 3.2. Folate Content in Guavas

A selection of guavas (Myrtaceae, *Psidium*) of various origins, among them feijoa (*feijoa sellowiana*), also named pineapple guava, were analyzed for their folate profiles (Table 2). The different guavas were analyzed unpeeled and showed similar folate contents (43.1 ± 5.16 µg/100 g–47.9 ± 0.57 µg/100 g). Since all guavas are eaten peeled and unpeeled, whole Australian grown feijoa fruits as well as pulp and peel were analyzed separately. The whole fruit appeared to have a folate content of 91.0 ± 1.98 µg/100 g, the pulp was lower in folate with 64.4 ± 2.57 µg/100 g, and the peel showed the highest content of 103 ± 4.32 µg/100 g. By contrast, the USDA states a folate content of 49 µg/100 g for guavas [23] and 23 µg/100 g for feijoas [24]. However, Akilanathan et al. [11] analyzed two different varieties of guavas and found also very different values ranging between 49.0 and 211 µg/100 g. The main vitamer in all guavas and feijoas analyzed was 5-CH_3_-H_4_folate.

### 3.3. Folate Content in Papayas

Papayas (Caricaceae, *Carica papaya*) are popular fruits, originally coming from the American tropics, however, nowadays papayas are grown widely in the tropics and subtropics worldwide. We analyzed two papaya fruits as well as seeds and pulp separately. The results are shown in Table 3. The papaya fruits revealed total folate contents of 61.6 ± 3.01 µg/100 g and 90.7 ± 1.24 µg/100 g. The folate content of the seeds (25.6 ± 5.91 µg/100 g and 41.2 ± 1.91 µg/100 g) was lower than that of the pulp (56.3 ± 1.48 µg/100 g and 90.8 ± 1.91 µg/100 g). Since the percentage share of seeds is very small compared to the pulp, the folate content of the pulp did not differ from the folate content of the whole fruit. Compared with different varieties analyzed in previous studies, our results are substantially higher. Akilanathan et al. [11] analyzed two different papaya varieties using microbiological assays and only found 11.0 µg/100 g and 23.0 µg/100 g. In the USDA (United States Department of Agriculture) data base, papaya is listed with a folate content of 37.0 µg/100 g [25]. The discrepancy of the analyzed folate content with Akilanathan et al. [11] can possibly be traced back to the differing determination method. The latter group used a microbiological assay with PteGlu as calibration standard. As already discussed in the introduction usually there are no significant differences between the quantification methods, but the accuracy of the microbiological assay can vary with the chosen calibrant [26]. As the main vitamer in papayas was 5-CH_3_-H_4_folate, PteGlu as a calibrant might have led to inaccuracies. Furthermore, an incomplete deconjugation could have led to underrated results, which we can exclude as we automatically test for deconjugation efficiency in each run. Apart from the methodological differences, the geographical origin and the different varieties can also be responsible for the inequality. Regarding the vitamer distribution, 5-CH_3_-H_4_folate was again the main vitamer in both fruits.

### 3.4. Folate Content in Jack Fruit

Jack fruit (Moraceae, *Artocarpus hereophyllus*), with its well flavored yellow pulp, is originally from India and is now indigenous in the tropics worldwide. The folate contents of two individual fruits were 83.6 ± 5.50 (1) µg/100 g and 52.9 ± 2.61 (2) µg/100 g, and therefore in a similar range to mangos (Table 4). Jack fruit seeds of fruit (1), which are embedded in the pulp and consisted of approximately around 10–15% of the total fruit had 51.1 ± 2.17 µg/100 g total folate. Furthermore, we analyzed commercially bought jack fruit chips and found 192 ± 3.38 µg/100 g total folate. The main vitamer in all analyzed jack fruit samples was 5-CH_3_-H_4_folate. The USDA specifies a total folate content for jack fruit of 24.0 µg/100 g and, consequently, we found a considerably higher folate content [27]. Akilanathan et al. [11] indicated a total folate content of 35 µg/100g, which is also a little lower than the analyzed content in the present study. As already discussed, the discrepancy in the total folate content can be caused by the different methods, the environmental impact and the variety of the fruits. Of note is the varying moisture content of the analyzed fruits compared to the literature, which can be caused by different ripening state or the different varieties. The moisture content of jack fruit (1) was 71.7% and that of jack fruit (2) was 79.7%, which may contribute to the rather high difference in folates of both fruits. In comparison to our samples, the moisture content of the jackfruit analyzed by Akilanathan et al. [11] was 76.0%, and stated by USDA as being 73.5% [27]. Due to the lack of information about the variety of the jackfruits, the reason of the different total folate content may only be assumed.

### 3.5. Folate Content in Other Tropical Fruits

The total folate content and vitamer profiles in a selection of mainstream and non-mainstream tropical fruits and vegetables is presented in Table 5. Three different pitayas (Cactaceae, *Hylocereus cacti*) were examined and total folate contents from 18.7 ± 0.11 µg/100 g, to 36.0 ± 0.53 µg/100 g were found. Chew et al. [9] also analyzed the folate content of commonly consumed Malaysian foods using microbiological assays and found a much lower folate content in dragon fruit of only 3 µg/100 g. A similar folate content of 23.8 ± 0.44 µg/100 g was found in prickly pear (Cactaceae, *Opuntia ficus-indica*). Salak (Arecaceae, *Salacca zalacca*), mainly grown in Asia but also in European Mediterranean regions, had a total folate content of 27.3 ± 2.09 µg/100 g. However, in a previous study, Salak was found to have a very low folate content of 6 µg/100 g [9]. The very popular fruits from the Longan-tree (Sapindaceae, *Dimorcarpus longan*) were also analyzed, having 67.8 ± 0.12 µg/100 g total folate. In contrast, Longkong (Meliaceae, *Aglaia dookoo*), which is present throughout South East Asia, appeared to be much lower in folate with only 15.9 ± 0.67 µg/100 g. Kaki (Ebenaceae, *Diospyros kaki*) can be eaten peeled or unpeeled. A folate content of 40.5 ± 1.33 µg/100 g and 50.5 ± 0.09 µg/100 g was found for peeled and unpeeled fruit, respectively. Chew et al. [9] analyzed a Korean persimmon (Pisang kaki) and again found a much lower folate content of only 6 µg/100 g, which is approximately seven times lower than our results. Lucuma (Sapotaceae, *Pouteria lucuma*), a fruit species mainly originating from South America, was found to have 41.8 ± 5.37 µg/100 g total folate. Since the fruit is eaten fresh or as flour, we calculated the total folate content also on a dry weight basis (209 ± 5.37 µg/100 g).

Several fruits belonging to the Passifloraceae family were also analyzed and found to be very high in folates. Among them, sweet granadilla (*Passiflora ligularis*) had a folate content of 64.0 ± 1.70 µg/100 g, passion fruit (*Passiflora edulis*) of 136 ± 21.7 µg/100 g, and yellow passionfruit (*Passiflora flavicarpa*) with 271 ± 3.64 µg/100 g was highest in total folate.

Tamarind (Fabaceae, *Tamarindus indica*) was quite low in folate with 11.4 ± 0.70 µg/100 g, whereas pete beans (Fabaceae, *Parkia speciosa*) were substantially higher with 100 ± 3.26 µg/100 g. The popular fruit Chinese jujube (Rhamnaceae, *Ziziphus jujuba*), mainly coming from China, had a folate content of 22.7 ± 0.23 µg/100 g. Cherimoya (Annonaceae, *Annona cherimola*) was found to have 48.4 ± 0.57 µg/100 g total folate. Two different batches of okras (Malvaceae, *Abelmoschus esculentus*), also known as Lady’s Finger, appeared to be a good natural source of folate with 101 ± 7.62 µg/100 g and 109 ± 3.91 µg/100 g, respectively. Okra as a good source of folate was already confirmed previously. Ismail et al. [10] found 100 µg/100 g total folate in okra analyzed by HPLC-UV. Devi et al. [28] found also a relative high folate content of 81 µg/100 g. Horned melons, also known as kiwano (Cucurbitaceae, *Cucumis metuliferus*), contained 7.82 ± 0.17 µg/100 g and 10.2 ± 0.31 µg/100 g total folates. The USDA listed horned melon as having a total folate content of 3.00 µg/100 g [29]. Furthermore, tamarillo, also known as tree tomato (Solanaceae, *Solanum betacea*), had a relative low folate content of 16.4 ± 0.60 µg/100 g.

5-CH_3_-H_4_folate was the main vitamer in most of the analyzed fruit and vegetable samples, except in salak, tamarind and one of the horned melon samples. The relative amounts of the individual vitamers (individual vitamer vs. total folate content in %) were as follows: 5-CH_3_-H_4_folate (14.9% to 94.8%), 5-CHO-H_4_folate (3.17% to 41.3%), 10-CHO-PteGlu (0.48% to 48.1%), H_4_folate (0.71% to 13.6%), and PteGlu (0.52% to 22.9%).

## 4. Conclusions

Total folate as well as the vitamer profile were analyzed in a broad range of tropical fruits and vegetables using LC-MS/MS and SIDA. The results clearly demonstrate that tropical fruits and vegetables can contribute to the daily supply with folate. Among the samples studied, varieties from passion fruit appeared to have the highest folate contents. It is also notable that longan fruits, okras, pete beans, papayas, mangos, jack fruits, and feijoas can be considered as good sources of folates. Particularly for some passion fruits such as *Passiflora flavicarpa*, a daily consumption of only 110 g would cover the recommended daily requirement for adults of 300 µg folate [30]. This content is surprisingly high for a tropical fruit. However, it cannot outcompete the folate concentration in the “king of fruits” Durian, with up to 440 µg/100 g total folate [31]. Moreover, several indigenous fruits and vegetables have not been analyzed before or have been analyzed using error-prone methods. To the best of our knowledge, lucuma, pitaya (red and yellow), longkong, prickly pear, longan, tamarind, pete beans, Chinese jujube, cherimoya, and tamarillo have been described for the first time regarding their total folate content. For all other fruits listed here, only scattered information about the total folate content is published. Being able to distinguish between the individual folate vitamers, LC-MS/MS and SIDA offer the most selective methods and are of choice for folate analysis. Due to the different stability of folate vitamers when exposed to light, heat, and oxygen, a known vitamer distribution can help to estimate the stability of folates in food. Assessing the absorption properties of different vitamers, high percentages of H_4_folate are lost during digestion. Consequently, fruits and vegetables containing high amounts of H_4_folate might be less bioaccessible [32]. The distribution of five common folate vitamers of the most fruits listed here are described for the first time.

In summary, this work presents an important overview of the total folate content and vitamer profiles in a broad range of tropical fruits and vegetables. Therefore, this work also generated crucial nutritional data about a broad range of indigenous fruits and vegetables. This is particularly the case for plant food originating from regions with high biodiversity like the tropics. Moreover, some of the fruits we have analyzed (e.g., feijoa) revealed very attractive sensory properties. However, as our study is far from being representative or covering the majority of tropical fruits, further bioprospecting, particularly on traditional “ethno food”, is necessary.

Moreover, we have not yet investigated the impact of maturity, climate, harvest season, and soil properties as well as pre- and post-harvest treatment, which can have a significant effect on the total folate content in fruits and vegetables. Therefore, follow-up studies taking these considerations into account are warranted.

## Figures and Tables

**Table 1:** Total folate content (µg/100 g, calculated as PteGlu) in mangos.

	5-CH_3_-H_4_folate	5-CHO-H_4_folate	10-CHO-PteGlu	H_4_folate	PteGlu	Total Folate
Mango varieties (*Magnifera indica*)						
Ataulfo (1)	67.7 ± 1.54	3.44 ± 0.07	0.84 ± 0.12	1.90 ± 0.18	0.57 ± 0.29	74.5 ± 2.09
(2)	61.2 ± 2.10	3.56 ± 0.07	0.57 ± 0.04	2.96 ± 0.30	0.82 ± 0.30	69.1 ± 1.96
Keith (3)	54.5 ± 1.24	3.84 ± 0.01	0.51 ± 0.03	1.56 ± 0.03	0.54 ± 0.00	60.9 ± 1.86
Thai-mango (4)	53.7 ± 0.08	3.64 ± 0.09	0.65 ± 0.02	1.63 ± 0.12	0.78 ± 0.27	60.4 ± 0.22
Palmer (5)	49.2 ± 1.02	2.97 ± 0.16	0.68 ± 0.13	1.69 ± 0.27	1.18 ± 0.06	55.8 ± 0.73

(1)–(5) were different mango varieties (Appendix A); data are means ± SD (n = 3).

**Table 2:** Total folate content (µg/100 g, calculated as PteGlu) in guavas.

	5-CH_3_-H_4_folate	5-CHO-H_4_folate	10-CHO-PteGlu	H_4_folate	PteGlu	Total Folate
feijoa (*Feijoa sellowiana*)						
peel	89.9 ± 3.46	5.56 ± 0.07	0.86 ± 0.42	5.74 ± 1.03	0.72 ± 0.08	103 ± 4.32
whole fruit	82.9 ± 2.31	4.02 ± 0.10	0.75 ± 0.10	2.96 ± 0.18	0.51 ± 0.15	91.0 ± 1.98
pulp	57.0 ± 1.13	4.27 ± 1.02	0.73 ± 0.09	1.67 ± 0.17	0.62 ± 0.26	64.4 ± 2.57
guava (*Psidium*)						
(1)	41.5 ± 0.50	1.31 ± 0.86	0.43 ± 0.26	1.39 ± 0.11	3.28 ± 0.08	47.9 ± 0.57
(2)	31.2 ± 1.16	8.13 ± 0.52	1.61 ± 0.07	2.36 ± 0.25	3.04 ± 0.00	46.3 ± 0.45
(3)	28.9 ± 3.14	7.20 ± 0.11	2.23 ± 0.40	1.99 ± 0.22	2.77 ± 0.00	43.1 ± 5.16

(1)–(3) are different guava varieties, data are means ± SD (n = 3).

**Table 3:** Total folate content (µg/100 g, calculated as PteGlu) in papayas.

	5-CH_3_-H_4_folate	5-CHO-H_4_folate	10-CHO-PteGlu	H_4_folate	PteGlu	Total folate
papaya (*Carica papaya*) varieties						
(1)	72.2 ± 0.48	5.62 ± 0.12	1.48 ± 0.18	11.4 ± 0.33	n.a.	90.7 ± 1.24
Formosa (2)	48.9 ± 0.89	3.79 ± 0.77	1.61 ± 0.23	6.95 ± 1.15	0.39 ± 0.07	61.6 ± 3.01
papaya pulp						
(1)	73.9 ± 0.56	3.81 ± 0.01	1.50 ± 0.01	11.6 ± 0.77	0.05 ± 0.00	90.8 ± 1.91
Formosa (2)	42.4 ± 0.49	4.40 ± 0.00	1.54 ± 0.09	7.62 ± 0.26	0.41 ± 0.00	56.3 ± 1.48
papaya seeds						
(1)	22.8 ± 0.38	9.90 ± 0.46	5.80 ± 0.40	2.06 ± 0.09	0.63 ± 0.06	41.2 ± 1.79
Formosa (2)	13.2 ± 2.42	6.38 ± 0.51	4.41 ± 0.72	1.28 ± 0.36	0.32 ± 0.16	25.6 ± 5.91

(1) and (2) are different papaya varieties, data are means ± SD (n = 3).

**Table 4:** Total folate content (µg/100 g, calculated as PteGlu) in jack fruits, jack fruit seeds, and jack fruit chips.

	5-CH_3_-H_4_folate	5-CHO-H_4_folate	10-CHO-PteGlu	H_4_folate	PteGlu	Total Folate
jack fruit (*Artocarpus hereophyllus*)						
chips	126 ± 0.00	18.1 ± 0.15	20.0 ± 0.80	2.97 ± 0.48	24.9 ± 1.95	192 ± 3.38
(1)	71.0 ± 4.48	9.15 ± 1.35	2.16 ± 0.38	1.05 ± 0.28	0.27 ± 0.13	83.6 ± 5.50
(2)	37.8 ± 1.09	3.85 ± 0.76	4.64 ± 0.36	2.15 ± 0.11	0.81 ± 0.07	52.9 ± 2.61
seed (1)	31.4 ± 2.13	10.3 ± 1.81	8.77 ± 1.97	0.69 ± 0.35	n.a.	51.1 ± 2.17

(1) and (2) are different jack fruits varieties, data are means ± SD (n = 3).

**Table 5:** Total folate content (µg/100 g, calculated as PteGlu) in different tropical fruits and vegetables.

	5-CH_3_-H_4_folate	5-CHO-H_4_folate	10-CHO-PteGlu	H_4_folate	PteGlu	Total Folate
yellow passion fruit (*Passiflora flavicarpa*)	257 ± 0.14	8.57 ± 1.93	1.75 ± 0.05	1.92 ± 0.04	1.73 ± 0.69	271 ± 3.64
passion fruit (*Passiflora edulis*)	127 ± 14.0	5.29 ± 0.73	0.96 ± 0.16	1.38 ± 0.06	1.64 ± 0.46	136 ± 21.7
okra (*Abelmoschus esculentus*)	87.1 ± 3.55	13.3 ± 0.33	3.42 ± 0.18	4.31 ± 1.89	0.94 ± 0.40	109 ± 3.91
okra (*Abelmoschus esculentus*)	76.4 ± 7.63	9.19 ± 0.33	11.5 ± 1.92	0.99 ± 0.13	2.41 ± 0.37	101 ± 7.62
pete beans (*Parkia speciose*)	70.2 ± 2.74	15.8 ± 1.40	5.65 ± 0.19	6.67 ± 0.23	1.66 ± 0.25	100 ± 3.26
longan (*Dimorcarpus longan)*	60.9 ± 0.37	3.76 ± 0.34	0.32 ± 0.12	2.47 ± 0.06	0.40 ± 0.08	67.8 ± 0.12
sweet granadilla (*Passiflora ligularis*)	52.4 ± 0.14	7.24 ± 0.72	1.58 ± 0.17	1.81 ± 0.06	0.98 ± 0.11	64.0 ± 1.70
kaki *(Diospyros kaki)* unpeeled	41.1 ± 0.10	3.77 ± 0.06	3.73 ± 0.06	1.47 ± 0.08	0.40 ± 0.24	50.5 ± 0.09
cherimoya (*Annona cherimola*)	23.8 ± 1.95	19.5 ± 2.63	2.57 ± 0.17	0.41 ± 0.25	2.11 ± 0.18	48.4 ± 0.57
lucuma *(Pouteria lucuma)*	32.7 ± 1.10	5.66 ± 3.52	0.46 ± 0.16	1.95 ± 0.17	1.11 ± 0.66	41.8 ± 5.37
kaki *(Diospyros kaki)* peeled	30.9 ± 0.31	5.73 ± 0.31	2.25 ± 0.10	1.53 ± 0.03	0.09 ± 0.01	40.5 ± 1.33
dragon fruit/pitaya (white) (*Hylocereus undatus)*	23.8 ± 0.29	4.90 ± 0.09	5.47 ± 0.00	1.61 ± 0.07	0.23 ± 0.07	36.0 ± 0.53
salak (*Salacca zalacca)*	8.24 ± 0.51	10.5 ± 1.05	3.42 ± 0.04	1.64 ± 0.21	3.49 ± 0.25	27.3 ± 2.09
prickly pear (*Opuntia ficus-indica)*	16.9 ± 0.50	3.39 ± 0.30	1.16 ± 0.35	0.88 ± 0.10	1.49 ± 0.15	23.8 ± 0.44
pitaya (red) (*Hylocereus costaricensis)*	13.0 ± 0.81	5.45 ± 0.90	3.74 ± 0.70	1.10 ± 0.10	0.50 ± 0.04	23.8 ± 0.88
chinese jujube (*Ziziphus jujuba*)	7.79 ± 0.20	4.45 ± 0.23	5.57 ± 0.83	0.32 ± 0.18	4.52 ± 0.56	22.7 ± 0.23
pitaya (yellow) (*Hylocereus megalanthus)*	10.7 ± 0.18	3.83 ± 0.04	3.09 ± 0.26	0.85 ± 0.01	0.26 ± 0.02	18.73 ± 0.11
tamarillo (*Solanum betacea*)	12.2 ± 0.53	2.23 ± 0.06	0.64 ± 0.04	0.91 ± 0.00	0.49 ± 0.00	16.4 ± 0.60
longkong *(Aglaia dookoo)*	9.39 ± 0.38	2.73 ± 0.14	1.00 ± 0.34	2.02 ± 0.07	0.81 ± 0.30	15.9 ± 0.67
tamarind (*Tamarindus indica*)	1.69 ± 0.24	1.24 ± 0.09	5.48 ± 0.76	0.37 ± 0.39	2.61 ± 0.15	11.4 ± 0.70
horned melon (*Cucumis metuliferus*)	3.30 ± 0.02	4.21 ± 0.05	1.05 ± 0.06	0.84 ± 0.15	0.82 ± 0.06	10.2 ± 0.31
horned melon (*Cucumis metuliferus*)	5.38 ± 0.07	1.34 ± 0.17	n.a.	1.06 ± 0.07	0.04 ± 0.02	7.82 ± 0.17

Data are means ± SD (n = 3).

## Appendix A

**Table A1:** List of countries of origin and number of fruits analyzed of all fruits listed in the publication.

Fruit	Country of Origin (Variety)	Number of Fruits Analyzed
guava (*Psidium*) (1)	Columbia	1
guava (*Psidium*) (2)	Singapore	1
guava (*Psidium*) (3)	Vietnam	1
feijoa (*Feijoa sellowiana*)	Australia	6
mango (*Magnifera indica*) (1)	Mexico (variety Ataulfo)	1
mango (*Magnifera indica*) (2)	Mali	1
mango (*Magnifera indica*) (3)	Puerto Rico (variety Keith)	1
mango (*Magnifera indica*) (4)	Thailand (variety Thaimango)	1
mango (*Magnifera indica*) (5)	Brazil (variety Palmer)	1
papaya (*Carica papaya*) (1)	Columbia	1
papaya (*Carica papaya*) (2)	Brazil (variety Formosa)	1
jack fruit (*Artocarpus hereophyllus*) (1)	Singapore	1
jack fruit (*Artocarpus hereophyllus*) (2)	Singapore	1
pitaya (red) (*Hylocereus costaricensis*)	Vietnam	1
pitaya (yellow) (*Hylocereus megalanthusi*)	Columbia	1
Dragon fruit /pitaya (white) (*Hylocereus undatus*)	unknown	1
prickly pear (*Opuntia ficus-indica*)	unknown	1
salak (*Salacca zalacca*)	Bali	1
longan (*Dimorcarpus longan*)	Vietnam	1
longkong (*Aglaia dookoo*)	Thailand	1
kaki (*Diospyros kaki*) peeled	unknown	1
kaki (*Diospyros kaki*) unpeeled	South Africa	1
lucuma (*Pouteria lucuma*)	Columbia	1
sweet granadilla (*Passiflora ligularis*)	unknown	1
passion fruit (*Passiflora edulis*)	unknown	1
yellow passion fruit (*Passiflora flavicarpa*)	unknown	1
tamarind (*Tamarindus indica*)	unknown	several
pete beans (*Parkia speciose*)	unknown	several
chinese jujube (*Ziziphus jujuba*)	unknown	1
cherimoya (*Annona cherimola*)	unknown	1
okra (*Abelmoschus esculentus*)	Singapore	several
okra (*Abelmoschus esculentus*)	Thailand	several
horned melon (*Cucumis metuliferus*)	unknown	1
horned melon (*Cucumis metuliferus*)	unknown	1
tamarillo (*Solanum betacea*)	unknown	1
